# Epigenetic mapping of the *Arabidopsis* metabolome reveals mediators of the epigenotype-phenotype map

**DOI:** 10.1101/gr.232371.117

**Published:** 2019-01

**Authors:** Rik Kooke, Lionel Morgado, Frank Becker, Henriëtte van Eekelen, Rashmi Hazarika, Qunfeng Zheng, Ric C.H. de Vos, Frank Johannes, Joost J.B. Keurentjes

**Affiliations:** 1 Laboratory of Genetics, Wageningen University and Research, 6708 PB Wageningen, The Netherlands;; 2 Laboratory of Biometris, Wageningen University and Research, 6708 PB Wageningen, The Netherlands;; 3 Centre for Biosystems Genomics, 6708 PB Wageningen, The Netherlands;; 4 Groningen Bioinformatics Centre, University of Groningen, 9747 AG Groningen, The Netherlands;; 5 Business Unit Bioscience, Wageningen Plant Research, 6708 PB Wageningen, The Netherlands;; 6 Institute for Advanced Study, Technical University of Munich, 85748 Garching, Germany;; 7 Tea Research Institute, Chinese Academy of Agricultural Sciences, 310008 Hangzhou, P.R. China;; 8 Netherlands Metabolomics Centre, 2333 CC Leiden, The Netherlands;; 9 Population Epigenetics and Epigenomics, Department of Plant Sciences, Technical University of Munich, 85354 Freising, Germany

## Abstract

Identifying the sources of natural variation underlying metabolic differences between plants will enable a better understanding of plant metabolism and provide insights into the regulatory networks that govern plant growth and morphology. So far, however, the contribution of epigenetic variation to metabolic diversity has been largely ignored. In the present study, we utilized a panel of *Arabidopsis thaliana* epigenetic recombinant inbred lines (epiRILs) to assess the impact of epigenetic variation on the metabolic composition. Thirty epigenetic QTL (QTL^epi^) were detected, which partly overlap with QTL^epi^ linked to growth and morphology. In an effort to identify causal candidate genes in the QTL^epi^ regions and their putative *trans*-targets, we performed in silico small RNA and qPCR analyses. Differentially expressed genes were further studied by phenotypic and metabolic analyses of knockout mutants. Three genes were detected that recapitulated the detected QTL^epi^ effects, providing evidence for epigenetic regulation in *cis* and in *trans*. These results indicate that epigenetic mechanisms impact metabolic diversity, possibly via small RNAs, and thus aid in further disentangling the complex epigenotype-phenotype map.

Due to their sessile nature, plants have developed an incredible chemical arsenal to fight disease and stress, attract pollinators, and interact with all kinds of organisms above and below ground (Allwood et al. 2008; Kegge and Pierik 2010). This diverse array of chemicals is manifested mostly in secondary metabolites, which are, compared to primary metabolites, more diverse, more tissue- and development-specific, and more involved in response to changes in the biotic and abiotic environment (Kooke and Keurentjes 2012). The plant secondary metabolic profile is easily adjustable and highly plastic, which is one of the reasons why plants can thrive in nearly all terrestrial habitats.

The accumulation of secondary metabolites in specific plant tissues enables a balanced division of resources that contributes to increased fitness and competitive ability (Kliebenstein et al. 2005). Flowers form the basis of the sexual reproductive organs, and as such they are important organs for the plant to protect from herbivores and pathogens. Moreover, they serve very specialized functions, such as attracting pollinators and securing anthesis, further strengthening the need for specific chemical compounds. In this respect, it is not surprising that flowers have a much more complex metabolic profile than vegetative tissues and that defense compounds are most concentrated in the reproductive organs of plants (Brown et al. 2003; Smallegange et al. 2007; Matsuda et al. 2010).

Because of adaptation to various biotic and abiotic environments, extensive natural variation in phytochemical profiles exists between and within species, which can be investigated to unravel the underlying regulation of secondary metabolism (Keurentjes 2009). The combination of genetic mapping populations with the (un)targeted analysis of large numbers of metabolites has revealed strong genetic regulation, both qualitatively and quantitatively (Kliebenstein et al. 2001b; Keurentjes et al. 2006; Chan et al. 2010, 2011; Schilmiller et al. 2010). Different metabolites within the same pathway can be regulated simultaneously by regulatory genes in *trans* or in *cis* by specific quantitative trait loci (QTL) (Kliebenstein et al. 2001a,b; Keurentjes et al. 2006).

Although the genetic basis of secondary metabolite variation is becoming better understood, the role of epigenetics in secondary metabolism has so far been largely overlooked. Epigenetic variation that causes phenotypic diversity has been identified in plants and can be successfully transmitted to offspring for several generations, providing evidence for epigenetic inheritance (Cubas et al. 1999; Manning et al. 2006; Johannes et al. 2009; Martin et al. 2009; Reinders et al. 2009; Cortijo et al. 2014; Kooke et al. 2015). Epigenetic variation is widespread and heritable in nature where it maintains independently of or in dependence on DNA sequence variation (Vaughn et al. 2007; Schmitz et al. 2013; Kawakatsu et al. 2016; Taudt et al. 2016). Only epigenetic variation that is maintained independently of genetic variation may be called pure epigenetic variation, but examples of such inheritance are scarce (e.g., Cubas et al. 1999; Manning et al. 2006; Silveira et al. 2013). Nonetheless, spontaneous epi-mutations are frequently observed in *Arabidopsis* and, if fixed, may alter gene transcription or other cellular phenotypes (Becker et al. 2011; Schmitz et al. 2011; Van der Graaf et al. 2015). It is thus becoming increasingly clear that epigenetic mechanisms play an important role in developmental processes and that they may be of evolutionary significance (Vidalis et al. 2016). A number of studies have reported a role for epigenetics in the regulation of secondary metabolism. In *Arabidopsis*, mutants dysfunctional in small RNA biosynthesis have significantly reduced amounts of glucosinolates in their leaves both in controlled conditions and upon caterpillar feeding compared to the wild-type Col-0, and it is suggested that small interfering RNAs can alter gene expression through DNA methylation variation that is inherited over multiple generations (Rasmann et al. 2012). In addition, flavonoid biosynthesis gene transcription is induced or repressed depending on the methylation state in methylation mutants and F_1_ hybrids (Kurihara et al. 2008; Shen et al. 2012).

Epigenetic recombinant inbred lines (epiRILs) in *Arabidopsis* were especially designed to study the impact of heritable epigenetic variation on complex traits (Johannes et al. 2009), and epigenetic QTL mapping approaches have shown that specific differentially methylated regions (DMRs) in the epiRILs can affect complex traits (Cortijo et al. 2014; Kooke et al. 2015). However, little is known about the regulatory mechanisms that govern changes from the molecular epigenotypic level up to the plant phenotypic level. Shedding light on the epigenetic regulation of plant metabolism might aid in better understanding the role of epigenetics in regulating plant growth and development on the molecular level.

Therefore, we analyzed the metabolic profile of 96 epiRILs using untargeted LC-MS metabolomics of both rosette leaves and flower heads and associated the observed variation to epigenetic variation in DNA methylation. To gain further insight into the epigenotype-phenotype map, we explored the epigenetic mechanisms underlying the detected QTL^epi^ effects and tested two possible hypotheses: (1) Methylation variation proximal to genes is involved in secondary metabolite regulation in *cis*; and (2) methylation variation in the QTL^epi^ interval modifies the production of small RNAs that target genes in *trans*, leading to altered regulation of metabolic and morphological phenotypes. Supporting evidence for both hypotheses was obtained.

## Results

### Tissue-specific epigenetic variation in plant secondary metabolism

**Figure 1.**
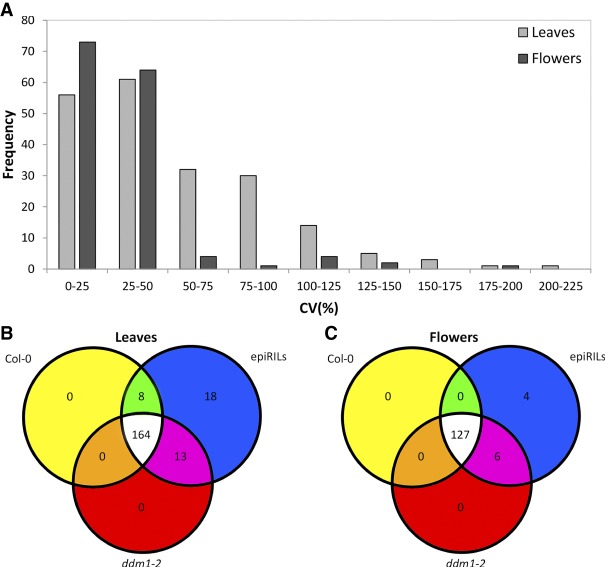
Metabolite variation in leaves and flowers of epiRIL population. (*A*) Frequency distribution of coefficient of variation (%) for all 203 leaf (light gray) and 149 flower (dark gray) metabolites detected in the Col-0 × *ddm1-2* epiRIL population using untargeted LC-QTOF-MS-based metabolomics. (*B*) Number of metabolites that were detected in the leaves of the parents of the population, Col-0 and *ddm1-2,* and the epiRILs. (*C*) Number of metabolites that were detected in the flowers of the parents of the population, Col-0 and *ddm1-2*, and the epiRILs.

To evaluate the effect of epigenetic variation on plant secondary metabolism, rosette leaves and flower heads from 96 epiRILs and their parents, Col-0 and *ddm1-2*, were analyzed by an LC-QTOF-MS-based metabolomics approach. In both tissues, qualitative and quantitative variation in metabolite accumulation could be observed among the epiRILs (Supplemental Tables S1, S2). In the leaves, 203 reconstructed metabolites could be retrieved. The observed variation in leaf metabolites was substantial (Fig. 1A) and very similar to the variation in conventional RIL populations (Supplemental Fig. S1; Keurentjes et al. 2006). The vast majority of leaf metabolites were detected in both parents and their derived epiRILs. However, a number of leaf metabolites were only detected in either one of the parents and a subset of the epiRILs (Fig. 1B). Besides qualitative and quantitative differences between the parents, 18 metabolites were identified that were solely detected in (a part of) the epiRIL population while being absent in both parents (Fig. 1B). These differences are either the result of epi-allelic transgressive segregation (Johannes and Colomé-Tatché 2011) that gives rise to the accumulation of novel metabolite structures, or alternatively, de novo epigenetic or genetic variation accumulated during the development of the epiRIL population.

In the flowers, 149 metabolites displaying substantial variation could be detected (Fig. 1A). As was the case for leaf tissue, the majority of metabolites in flowers was detected in both parents and their derived epiRILs, whereas a minority was, in addition to a limited number of epiRILs, only detected in Col-0 or *ddm1-2* flowers (Fig. 1C). Four metabolites were only detected in a portion of the epiRILs and not in the parents, suggesting that epi-allelic transgressive segregation has resulted in the accumulation of these metabolites. These findings indicate that epi-allelic variation can impact metabolic variation in a quantitative and qualitative manner in both flowers and leaves.

**Figure 2.**
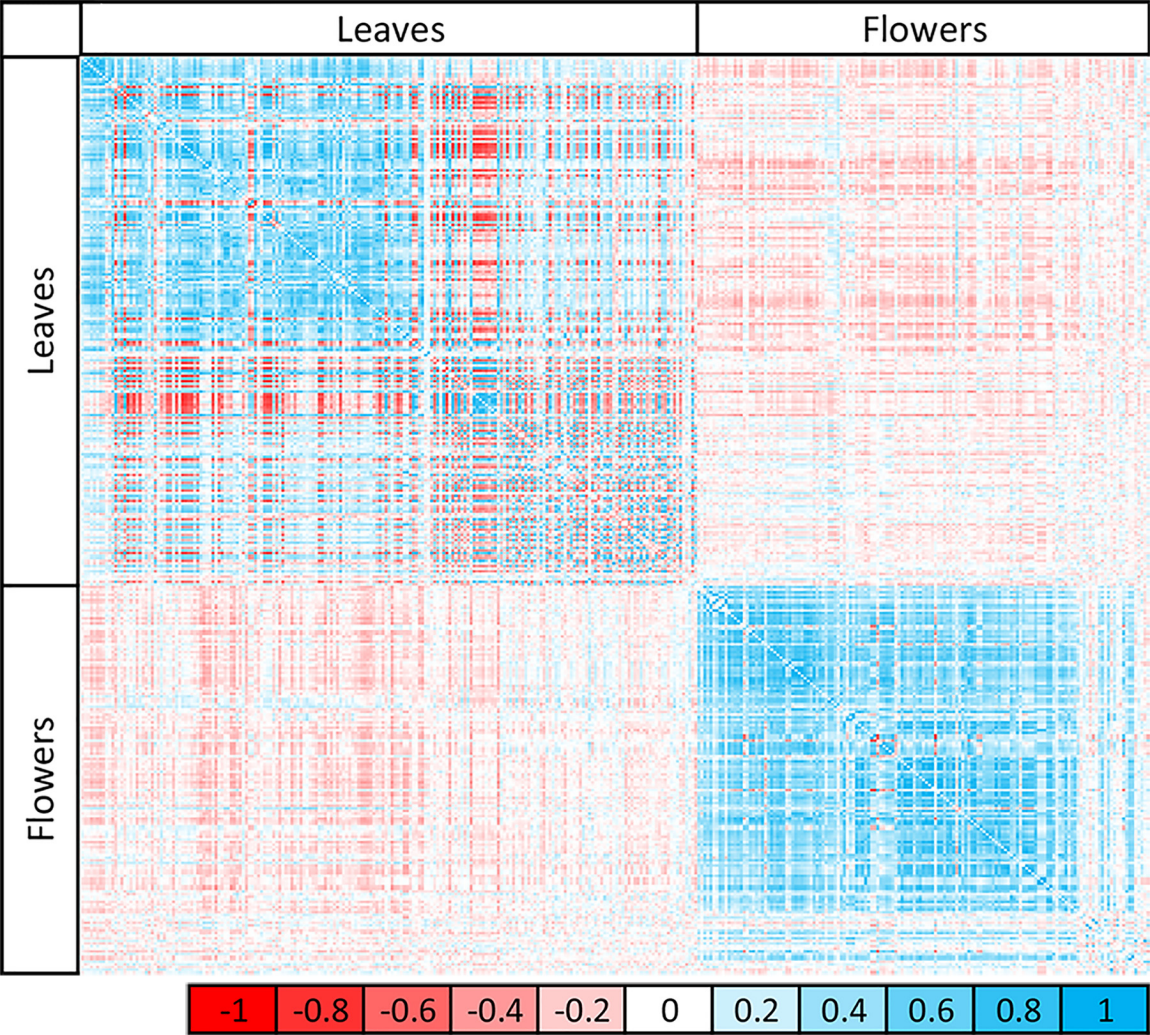
Correlation matrix of detected metabolites in the epiRIL population. Pearson's correlation between metabolites within and between tissues is indicated by color intensity from −1 (red) to 1 (blue). Variation in metabolites correlates within the same tissue, but correlation between different tissues is much weaker.

Strong correlations between metabolites across all epiRILs were detected within the same tissue, but much weaker correlations occurred between metabolites in different tissues (Fig. 2). Although the total number of correlating metabolites was quite similar in leaves and flowers (55% over 53%, respectively; ρ > 0.2), the proportion of negative correlations between metabolites was much higher in leaves than in flowers (46% over 8%, respectively) (Fig. 2), suggesting a stronger competition for resources in the leaves than in the flowers, possibly because of the dual role of leaves as both sink and source tissue. The high proportion of positive correlations in the flowers indicates that flowers show a much more coordinated regulation of metabolite accumulation, which might be caused by the tight developmental control and specific function of this tissue.

Although the leaf and flower tissues were not harvested from the same plant, some significant correlations (*P* < 0.05) between leaf and flower metabolites (10%, |ρ| > 0.2) could be observed, with the majority of them being negative (8.3%, ρ < −0.2) (Fig. 2). This illustrates the metabolic separation in tissue types and their functionally different roles in the plant's life cycle demanding distinct phytochemical profiles. The wide range of quantitative variation in metabolites between the WT Col-0 and *ddm1-2* parents of the population as well as between epiRIL individuals further suggests that the methylation status might be important for tissue-specific metabolic control.

### Site-specific differential methylation explains qualitative and quantitative metabolic variation

To gain deeper insight into the regulation of plant metabolism within the epiRIL population, QTL^epi^ analysis was performed on all metabolites using a genetic map based on differentially methylated regions as physical markers (Colome-Tatche et al. 2012). To verify that these DMRs were stably inherited to the generation of epiRILs used in our study, we subjected four randomly selected epiRILs to whole-genome bisulphite sequencing (Supplemental Fig. S2; Lauss et al. 2018). The detected methylation patterns in these four lines was remarkably similar to those reported earlier (Johannes et al. 2009; Colome-Tatche et al. 2012; Cortijo et al. 2014), demonstrating once more the stable inheritance of epigenetic marks over many generations.

**Figure 3.**
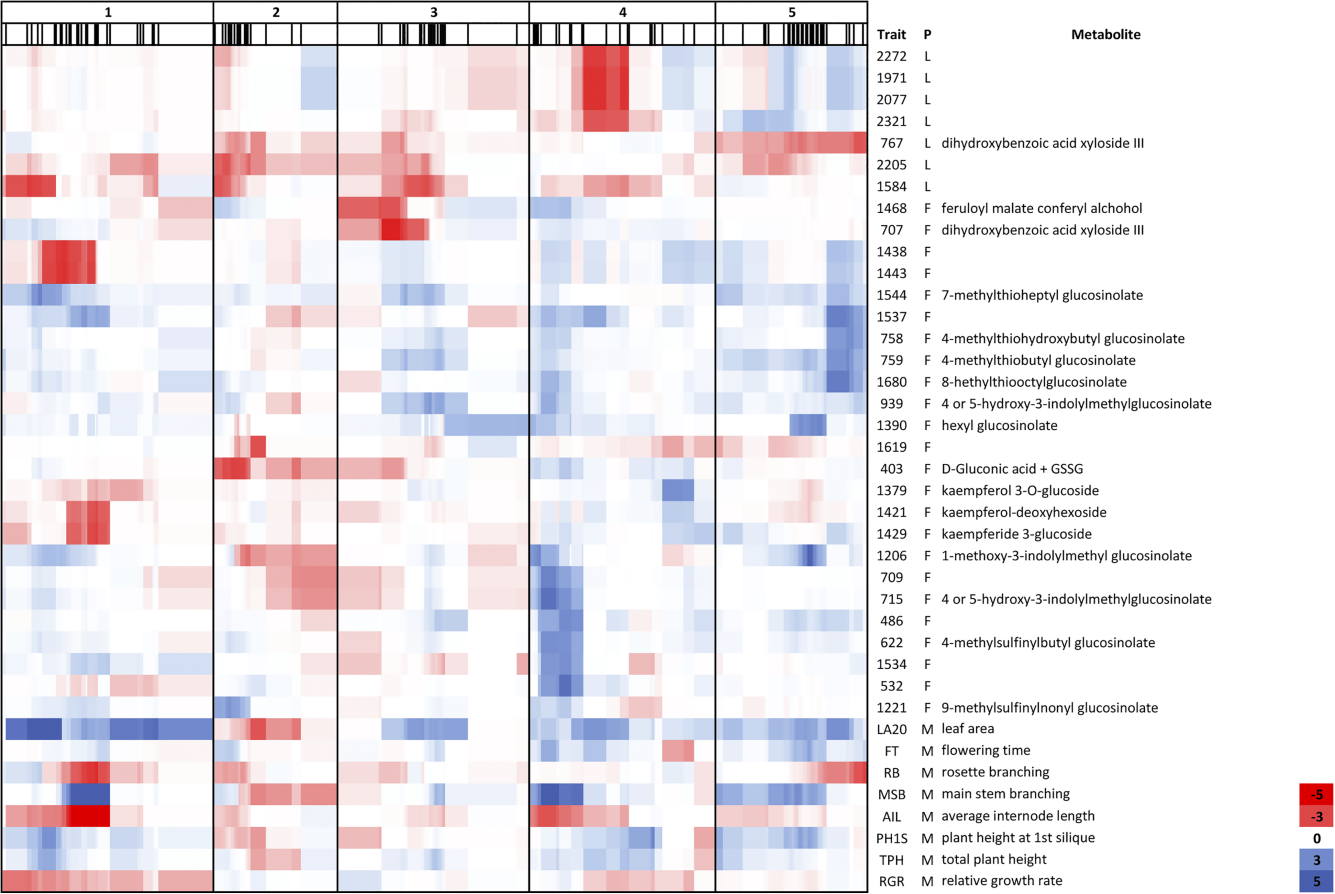
QTL^epi^ heat map for metabolic and morphological traits. QTL^epi^ heat map showing the positions of the QTL^epi^ and the overlap with QTL^epi^ for morphological traits divided over the five chromosomes. The morphological traits were described previously (Kooke et al. 2015). The thin black lines in the *second* row indicate the marker positions in cM. (Trait) Metabolite number or morphological trait, (P) phenotype group, (L) leaf, (F) flower, (M) morphology. The legend on the *right* indicates the QTL LOD score between −5 (red) and 5 (blue).

In total, 34 QTL^epi^ were identified for 30 different metabolites (Fig. 3; Supplemental Table S3). The widespread quantitative and qualitative variation that was detected in the epiRILs was reflected in the detected QTL^epi^. For example, QTL^epi^ were identified for metabolites that showed qualitative or quantitative variation between the parents of the epiRIL population. In addition, QTL^epi^ could be detected for metabolites with similar abundance in the two parents, indicating that transgressive segregation of the epigenetic markers within the population is probably responsible for the mapped metabolic variation in these epiRILs.

Out of the 34 QTL^epi^, 10 QTL^epi^ were detected in the leaves and 24 in the flowers. The epigenetic variation resulted in increased or decreased metabolite content depending on the metabolite and the tissue. Sixteen of the 34 QTL^epi^ displayed a negative effect sign, representing an increase in metabolite content between 4% and 41% in the *ddm1-2*-inherited epigenotypes. This was true for nine of the 10 QTL^epi^ detected for leaf metabolites, while this was the case for only eight of the 24 QTL^epi^ detected in the flowers. Overall, the detection of QTL^epi^ suggests that the observed metabolic variation among the epiRILs can at least partly be explained by methylation variation at DMRs.

### Epigenetic variation exerts pleiotropic effects on molecular and morphological traits

Twenty-one different QTL^epi^ regions could be assigned, divided over the five chromosomes, with many coinciding QTL^epi^ (Fig. 3; Supplemental Table S3). One QTL^epi^ region was shared between leaf and flower metabolites, while five regions were specific for leaf metabolites and 15 for flower metabolites. For most of the annotated compounds, QTL^epi^ could only be detected in one specific tissue, predominantly in flowers. However, for a limited number of metabolites, different QTL^epi^ were identified in leaves and flowers, indicating differential metabolic regulation between tissues (Supplemental Table S3). Altogether, these QTL^epi^ analyses suggest that epigenetics might play a significant role in regulating the tissue-specific accumulation of secondary metabolites.

The metabolic QTL^epi^ identified in this study overlapped with the morphological QTL^epi^ that were analyzed in the same experiment (Kooke et al. 2015) and with morphological QTL^epi^ detected in a previous study (Fig. 3; Cortijo et al. 2014). Twelve pleiotropic QTL^epi^ regions were detected, divided over the five chromosomes but with especially strong pleiotropic loci in the middle of Chr 1 and 4, the start of Chr 4, and the middle and lower arm of Chr 5. The majority of metabolites for which an QTL^epi^ could be detected significantly correlated with the morphological traits that mapped to the same regions (*P*[*χ*2] < 0.01). The highest correlation was detected between flowering time and kaempferol-deoxyhexoside (*r* = −0.43), while both mapped to the same DMR on Chr 1 (Fig. 3; Supplemental Table S4). This suggests that metabolites are connected to morphological traits and that they might be regulated by the same epigenetic mechanisms.

Alternatively, the variation in metabolites might be a pleiotropic effect of differences in flowering time, although the sampling of flowers for metabolic profiling was such that each individual flower head was harvested at the time when the first flower opened. Nonetheless, variation in a number of flower and leaf metabolites that significantly associated with a DMR demonstrated a significant correlation (*P* < 0.001) with flowering time (Supplemental Table S4). Therefore, the QTL^epi^ analysis was also applied with metabolic values corrected for variation in flowering time. This analysis revealed a very similar pattern with only slight differences in the number of detected QTL^epi^, mostly due to threshold effects (Supplemental Table S5). Only two metabolites had a markedly changed QTL^epi^ profile upon correction for flowering time (Supplemental Fig. S3). It thus appears that for only a limited number of cases flowering time has a significant effect on the QTL^epi^ profile but that the majority of detected metabolic QTL^epi^ are not due to pleiotropic effects of the variation in flowering time.

### Regulation of secondary metabolism in *cis* by epigenetic variation in biosynthesis genes

To investigate epigenetically regulated candidate genes involved in secondary metabolism, we focused our attention on variation in glucosinolate and flavonoid content of the flowers. Sixty-seven candidate genes were selected within the 1.5 LOD QTL^epi^ confidence intervals, based on their involvement in glucosinolate and/or flavonoid metabolism according to the TAIR, ARACYC, and KEGG databases (Berardini et al. 2015; Kanehisa et al. 2017; Schläpfer et al. 2017). We next submitted each gene to a series of strict selection criteria. For all 67 genes, differentially methylated regions in the promoter, gene body, and 1 kb downstream from the candidate gene in the epiRILs were associated to their metabolic trait values. For 27 out of 67 genes, significant (*P* < 0.05) associations were detected between methylation state and metabolic level. Because methylation states can be gained and lost, independent of the crossing scheme, it was investigated whether the methylation state at the DMR cosegregated with the most significant marker from the QTL^epi^ study to determine whether the DMR of the candidate gene can explain the QTL^epi^ (*P* < 0.05). This was the case for 17 of the 27 remaining genes. From these 17 genes, we selected nine candidates based on the relationship between gene function and metabolite pathway, position of the DMRs (promoter > gene body > downstream), presence of TEs close to DMRs, and gene expression variation between Col-0 and *ddm1-2* in publicly available data (Supplemental Table S6; McCue et al. 2013; Stroud et al. 2013).

**Figure 4.**
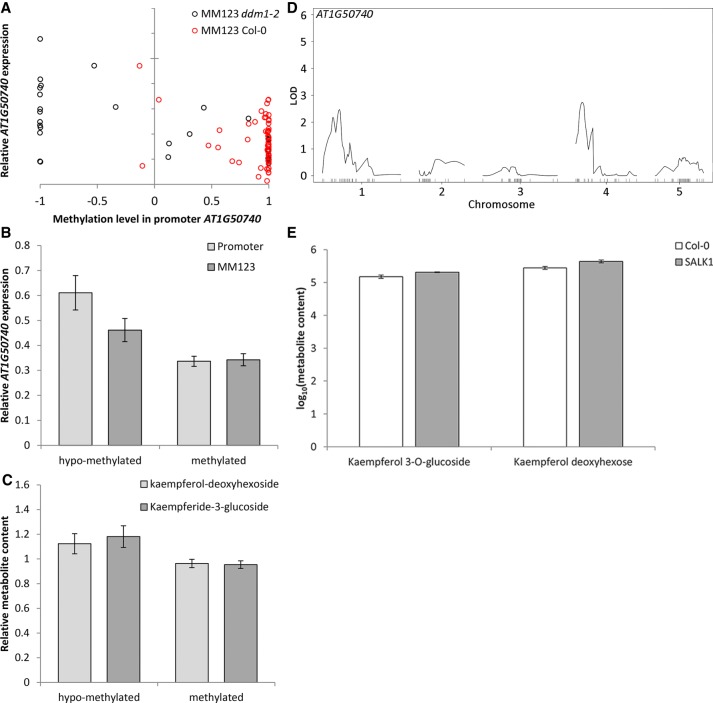
Confirmation analyses for *AT1G50740*. Methylation variation in the promoter of *AT1G50740* is associated with variation in gene expression and metabolite content. (*A*) Scatterplot indicating the correlation between methylation at promoter and gene expression of *AT1G50740* in all epiRILs. Red circles indicate epiRILs with wild-type allele at the DMR MM123; black circles indicate epiRILs with *ddm1-2* allele at DMR MM123. (*B*) Histograms indicating the association of the methylation level at the promoter of *AT1G50740* (light gray) and DMR MM123 (dark gray) with the relative gene expression of *AT1G50740*. (*C*) Histograms indicating the association of the methylation level at promoter of *AT1G50740* with the relative metabolite content of kaempferol-deoxyhexoside (light gray) and kaempferide-3-glucoside (dark gray). Hypomethylated indicates a methylation level between −1 and −0.3; methylated indicates a methylation level between −0.3 and 1. (*D*) eQTL^epi^ analysis for *AT1G50740* in epiRILs. (*E*) Variation in metabolite content of kaempferol-3-O-glucoside and kaempferol deoxyhexoside in wild-type Col-0 and *AT1G50740* knock-out mutant (designated SALK1 here; see Supplemental Material for details).

To determine whether the epigenetic variation was associated with variation in gene expression, qPCRs were performed on these nine genes in all epiRILs. Only one gene, *AT1G50740*, displayed a significant effect of both the DMR marker and the methylation levels around the gene on the gene expression levels (*P* < 0.05) (Fig. 4A,B). Specifically, nine DMRs in the promoter region of *AT1G50740* were significantly associated with variation in gene expression and the metabolic levels of two flavonoids that were associated with the QTL^epi^ (Fig. 4C). In addition, expression QTL^epi^ (eQTL^epi^) analyses suggest that the expression of *AT1G50740* might be affected by the methylation state at different loci (Fig. 4D). *AT1G50740* is a transmembrane protein possibly involved in defense responses and in the regulation of flavonoid biosynthetic processes (Heyndrickx and Vandepoele 2012). Epigenetic variation in the gene body and promotor of this gene was also observed in natural accessions of *Arabidopsis* (http://neomorph.salk.edu/1001.aj.php) (Supplemental Fig. S4), indicating that epigenetic variation in this gene is not just an experimental artifact but might also contribute to natural variation.

To further elucidate the involvement of *AT1G50740* in secondary metabolism, a knock-out mutant was analyzed using deep phytochemical phenotyping (Supplemental Table S7; Supplemental Fig. S5). Indeed, the comparison of the knock-out mutant of *AT1G50740* with the Col-0 WT revealed strong effects of this gene on the levels of several flavonoids (*P* < 0.05) (Supplemental Table S7; Fig. 4E). These findings indicate that methylation in the promoter of *AT1G50740* might regulate gene expression and flavonoid content.

### Regulation of secondary metabolism and plant morphology in *trans* by epigenetic variation in putative small RNAs

**Figure 5.**
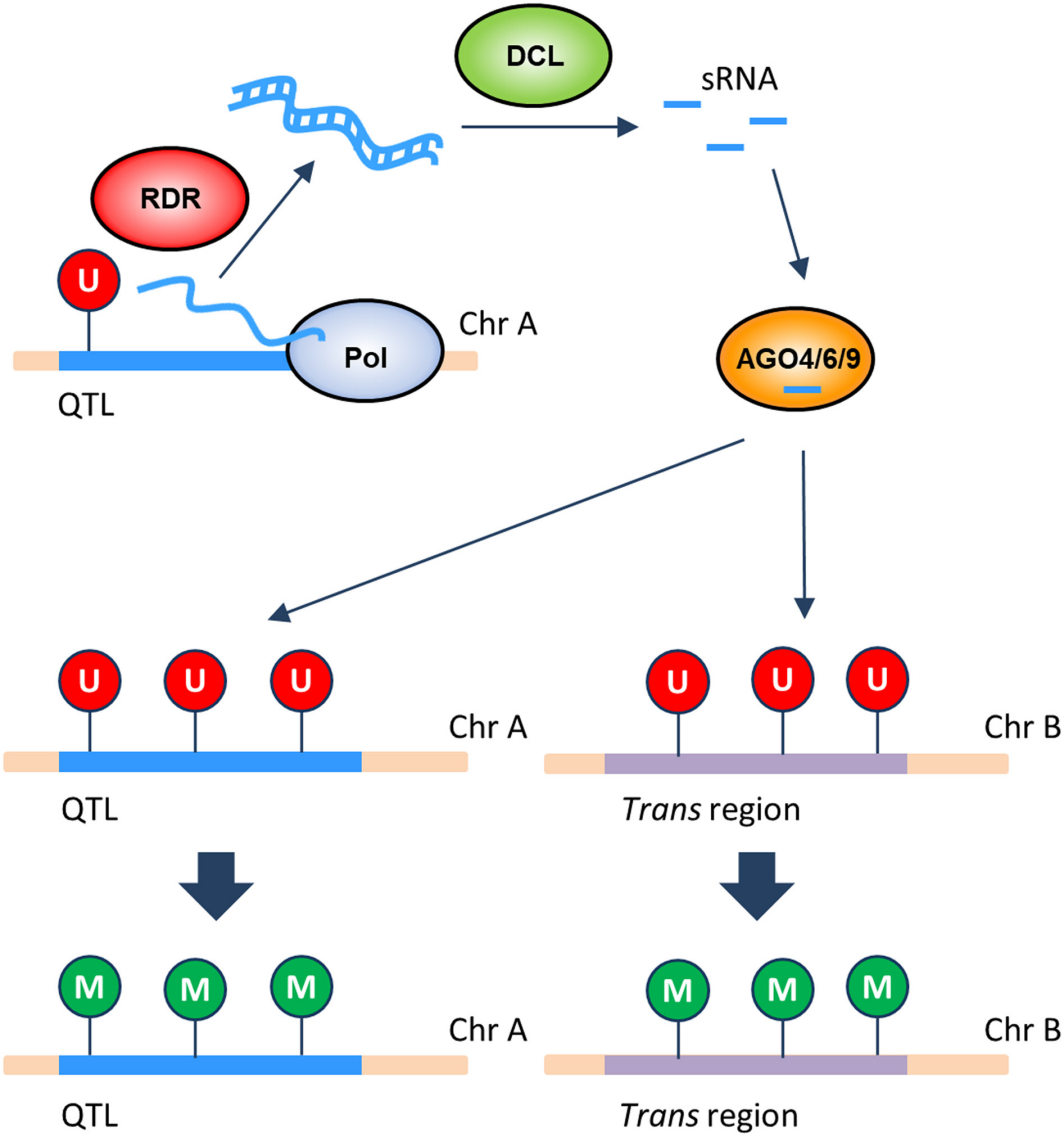
Theoretical model for the regulation of DNA methylation by differential targeting of sRNA to loci in *trans*. Changes in DNA methylation can be induced directly by differential recruitment of components of the RdDM pathway, or indirectly by post-transcriptional silencing of genes. (DCL) Dicer, (M) methylated, (Pol) RNA polymerase, (RDR) RNA-dependent RNA polymerase, (U) unmethylated.

Exploratory analyses revealed that QTLs^epi^ are also associated with DNA methylation states at promoter regions of 324 genes in *trans* (Supplemental Table S8). One molecular model that could explain these associations is that TEs or repeat-associated DMRs in QTL^epi^ intervals lead to the differential production of small RNAs that affect DNA methylation maintenance at loci in *cis* but also possibly in *trans* via the canonical or noncanonical RNA-directed DNA-methylation (RdDM) pathways (Matzke and Mosher 2014). Differential targeting of sRNA to loci in *trans* could induce DNA methylation changes either directly by altering the recruitment of components of the RdDM pathway, or indirectly by post-transcriptional silencing of genes flanking the *trans*-target loci (Fig. 5).

Although this hypothetical mechanism is difficult to validate experimentally, a key requirement is that regions in the QTL^epi^ intervals have sequence similarity with their putative *trans*-targets and that these target sequences match functional sRNA. To evaluate this, we searched the promoters of the 324 genes for segments sharing perfect similarity with their associated QTL^epi^. The homologous regions were then decomposed, in silico, into sets of artificial sRNAs (artsRNAs). Selected artsRNAs were then submitted to the SAILS computational framework (Morgado et al. 2017) to predict loading into ARGONAUTE (AGO) proteins 4/6/9, which are known to be involved in transcriptional silencing in plants (Fang and Qi 2016). ArtsRNA with high probability for AGO-loading were matched to true sRNAs from wild-type (WT) and *ddm1* sRNA libraries (Slotkin et al. 2009) to obtain further evidence to support these segments as real sRNAs (Supplemental Table S9).

**Figure 6.**
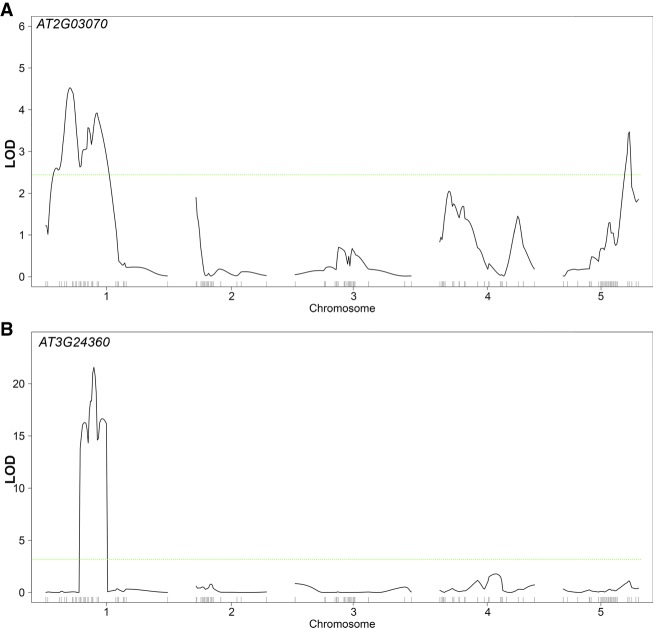
Expression QTL analysis in epiRILs. (*A*) *AT2G03070* and (*B*) *AT3G24360*. RNA was extracted for 93 epiRILs, reverse-transcribed to cDNA, and quantified by SYBR Green qPCR. Gene expression was normalized against the reference gene *TIP41* and subjected to eQTL^epi^ analyses. Green line indicates LOD significance threshold that was calculated using 1000 random permutations with α 0.05 as the genome-wide type 1 error level. Markers positions are indicated on the *bottom* of the graph.

Thirteen of the potential artsRNA target genes were further analysed for gene expression variation in the flower heads of the epiRIL population using qPCR and subsequent eQTL^epi^ analysis. Three of the 13 genes were significantly associated with one or multiple eQTL^epi^. For *AT3G24360* and *MED8*, the detected eQTL^epi^ colocated with the *trans*-QTL^epi^ interval that contains artsRNAs predicted to mediate transcriptional silencing (Fig. 6A,B; Supplemental Table S9). In addition, *MED8* and *AT3G24360* contain TEs in their promoters and all artsRNAs originating from the QTL^epi^ interval are complementary to these TEs or their flanking sequences (<1000 bp). In the case of *AT3G24360*, TEs from VANDAL families are found in all candidate regions targeted by artsRNAs. VANDAL transposons from the MuDR superfamily in maize have been shown to modulate the expression of genes through epigenetic mechanisms (Kinoshita et al. 2004). Following this approach, we thus established a link between methylation variation in small RNAs and *trans* genes and their level of expression.

**Figure 7.**
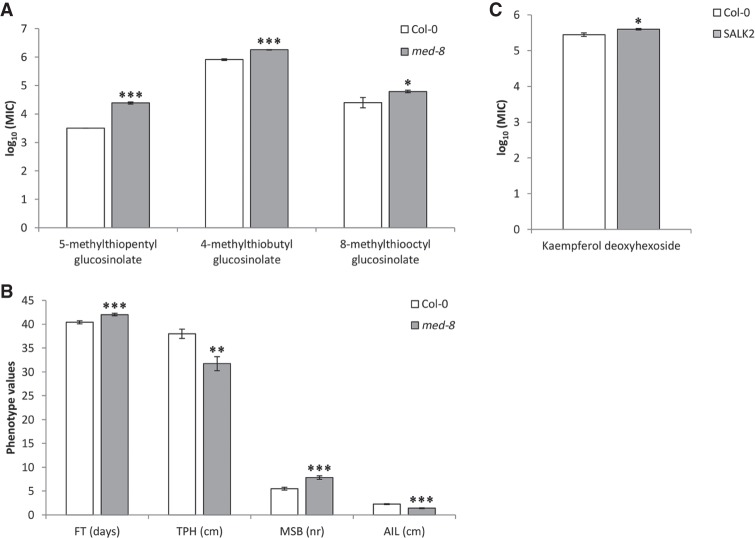
Metabolic and morphological trait analyses in mutants and wild type. (*A*) Metabolic values for three different glucosinolates (4-methylthiobutyl glucosinolate, 5-methylthiopentyl glucosinolate, and 8-methylthiooctyl glucosinolate) in Col-0 wild type and the *med-8* mutant. (*B*) Phenotypic trait values for flowering time (FT), total plant height (TPH), main stem branching (MSB), and average internode length (AIL) in col-0 wild type and the *med-8* mutant. (*C*) Metabolic values for kaempferol deoxyhexoside in Col-0 wild type and the *AT3G24360* knock-out mutant (designated SALK2 here; see Supplemental Material for details). (*) *P* < 0.05, (**) *P* < 0.01, (***) *P* < 0.001.

To illustrate that loss of expression affects metabolic and morphological traits, the metabolite profiles of KO mutants for the genes *AT3G24360* and *MED8* were compared with the Col-0 wild type. Both knock-out mutants displayed a significant reduction or complete loss of expression (Supplemental Fig. S5). The functional *MED8* mutant was significantly altered in the levels of 70 metabolites, including various glucosinolates and flavonoids (*P* < 0.05) (Supplemental Table S7; Fig. 7A). These findings coincide well with the function of *MED8* in mediating cross-talk between glucosinolate and phenylpropanoid biosynthesis pathways (Kim et al. 2015). Several morphological features were significantly altered in the mutant as well (Fig. 7B). The function of the Mediator complex in plant metabolism suggests that *MED8* is directly involved in regulating glucosinolate variation, which, in turn, may also alter flowering time, as indicated by a number of independent studies (Atwell et al. 2010; Kerwin et al. 2011; Jensen et al. 2015), although flowering time variation may also alter glucosinolate levels (Mohammadin et al. 2017). Likewise, the functional knock-out mutant of *AT3G24360* significantly altered the levels of several metabolites (*P* < 0.05) (Fig. 7C; Supplemental Table S7).

In addition, using the methylation data from the 1001 Genomes Consortium (Kawakatsu et al. 2016), variation could be observed in the level of methylation in the promoters of the candidate genes in natural accessions (Supplemental Fig. S4). Comparison of these methylation data with mRNA expression data in a public methylation browser (neomorph.salk.edu/1001.aj.php) revealed that de-methylation of the promoter correlates with altered gene expression (Supplemental Fig. 6A–C). These findings again indicate that variation in the level of methylation might play a role in natural settings as well.

## Discussion

### Epigenetic regulation of plant secondary metabolism

The findings presented here indicate that epigenetics is at least partly involved in the regulation of plant secondary metabolism in both leaves and flowers of *Arabidopsis*. It must be noted, however, that, given the large variation among the epiRILs, the number and strength of QTL^epi^ was considerably lower than the strength and number of metabolic QTL in genetic studies on classical RILs (Keurentjes et al. 2006; Rowe et al. 2008). Although the populations that were used in those studies were substantially larger, the results indicate that genetic variation is a much larger source for metabolic variation than epigenetic variation in *Arabidopsis*. On the one hand, this is not surprising given the long evolutionary history of *Arabidopsis* and its genetic adjustment to different environments (Weigel 2012). On the other hand, different studies on morphological traits claim that epigenetic variation can have an almost as large effect on morphological variation as genetic variation (Johannes et al. 2009; Zhang et al. 2013; Cortijo et al. 2014; Kooke et al. 2015). Given the understanding of plant metabolism as a blueprint for plant morphology and development (Kooke and Keurentjes 2012), the low number of QTL^epi^ is counterintuitive. It might be that the epigenetic effects on a metabolic scale are too small to be identified in QTL^epi^ analyses. On a higher level, however, e.g., in the case of morphological changes that are the outcome of canonical changes in a large set of metabolites, epigenetic effects may play a role.

The epigenetic control of secondary metabolite content in terms of number and strength of QTL^epi^ was much stronger in flowers than in leaves. Flowers, as reproductive organs, are important plant tissues in terms of fitness and should thus be well protected (McCall and Irwin 2006). Given the high unpredictability of pathogen and herbivore attack, plastic, epigenetic responses might be an additional line of defense for adequate responses to a broad range of attackers in the highly specialized flowers. Indeed, metabolites such as glucosinolates and flavonoids that are involved in defense against biotic and abiotic stress (Shirley 1996; Graham 1998; Wentzell and Kliebenstein 2008) were detected to be under epigenetic control in flowers. Epigenetic variation is thought to be especially important in fluctuating environments because epigenetic variants, in contrast to DNA sequence variants, can be reversed (Rando and Verstrepen 2007). Epigenetic modifications can unlock phenotypic plasticity and, as such, can enhance adaptation in such environments (Zhang et al. 2013; Kooke et al. 2015).

Moreover, some metabolites were not present in the Col-0 WT but were present in a number of epiRILs and the *ddm1-2* mutant. Two of these metabolites were significantly associated to DMRs, and epigenetic regulation can thus initiate the production of additional metabolites. Although the methylation differences were artificially induced in the epiRILs, the production of other metabolites through epigenetic means could be an effective weapon against herbivore attack in nature, especially under changing conditions. The control of secondary metabolism in plants is thus partly regulated via epigenetic mechanisms, possibly as an adaptation to respond to different levels of abiotic and biotic stresses, enhancing phenotypic plasticity.

### Epigenetic regulation in *cis* and in *trans*

DNA methylation variation is evidently the most likely reason for the observed phenotypic variation in the epiRILs. Indeed, qPCR analysis of *cis*-regulated genes revealed that hypomethylation of the promoter increases the expression of the gene, and knock-out mutant analyses confirmed that those genes are involved in regulating the accumulation of metabolites. In addition, sets of high-confidence artsRNAs were found to map to both the QTL^epi^ interval and the promoter of *trans* genes targeting the methylation state of the loci. We further observed that, both in the QTL^epi^ and the gene promoters, the artsRNAs often mapped to TEs/repeats or to their vicinity in DMRs. This is a relevant observation, since TE/repeat rich regions are known to influence changes in DNA methylation not only at a local level but also in distant genomic loci exactly through the production of sRNAs (Lewsey et al. 2016). Moreover, some artsRNAs map in QTL^epi^ intervals to DMRs that show a tendency of reversion to WT-like methylation levels. More specifically, the loss of methylation in multiple loci is accompanied by a decrease in the production of heterochromatic small interfering RNA (hc-siRNA) that in some plants reach such low levels that the feedback loop that sustains the methylation marks must be disrupted, affecting the capacity of the plants to recover and thus keeping the QTL source region and the *trans* target gene promoter depleted of DNA methylation. qPCR confirmed that the expression of these genes is increased if their methylation is reduced. We further confirmed with knock-out mutants of such genes that a loss of expression causes significant variation in plant metabolism. These results confirm the findings of a study which reported on mutants impaired in small RNA biogenesis that have significantly reduced levels of various aliphatic and indole glucosinolates (Rasmann et al. 2012).

Finally, we were able to show that variation in the level of methylation exists in the promoters of candidate genes in natural accessions, which correlates well with gene expression variation. The methylation variation observed in the epiRIL population might thus be relevant in natural settings as well.

### Pleiotropy

It is intriguing that the majority of QTL^epi^ detected for morphological traits in control and stress conditions, phenotypic plasticity, and secondary metabolism can be collapsed into 12 QTL^epi^ regions (Cortijo et al. 2014; Kooke et al. 2015). It appears that the epigenetic variants underlying the QTL^epi^ affect many phenotypic traits in parallel. Although the number of phenotypes measured in these studies is much smaller than the number of traits analyzed in the genetically diverse Cvi x L*er* population, it seems that the effects are similar in terms of pleiotropy and robustness (Fu et al. 2009). The master epigenetic regulators are most likely sRNAs that became inactive through hypomethylation in the F_1_ and have contributed to the alteration of the methylation state at various loci in *trans* which have maintained that state through meiosis. Indeed, independent knock-out mutants of two small RNA target candidate genes were shown to have significant effects on plant metabolism and morphology. For instance, we detected strong differences in the content of so-called Arabidopsides, which contain esterified oxylipins that are precursors for the plant defense hormone jasmonic acid (Glauser et al. 2008; Göbel and Feussner 2009). Oxylipins are derived from the oxidation of polyunsaturated fatty acids, and the levels of these compounds in a knock-out mutant of a gene encoding fatty acid beta oxidation activity were up to 11 times higher compared to the wild type. The detected epigenetic regulation of this gene (*AT3G24360*) through small RNAs had a direct effect on the levels of oxylipin compounds in our study, and given that there is natural methylation variation at the promoter of this gene, the epigenetic effects can have important implications for plant defense in nature.

## Methods

### Plant growth conditions

Seventeen replicate plants per epiRIL and parent were completely randomly grown in a climate chamber. At 21 d after germination (DAG), six randomly selected replicates were harvested for leaf tissue. At the time of flowering, the flower head was harvested for six other randomly selected replicates (see Supplemental Material for details on growth conditions).

### KO analysis

Homozygous mutants were grown in a completely randomized design in the same conditions as the epiRILs. Flowering time was recorded at the opening of the first flower. For 15 replicates, flower heads were harvested. For the 12 remaining replicates, main stem branching (MSB), rosette branching, plant height at first silique (PH1S), total plant height (TPH), and average internode length (AIL) were measured 2 wk after flowering. Gene expression of KO lines was analyzed using qPCR (see Supplemental Material for details on phenotyping methods).

### LC-QTOF-MS analysis of leaf and flower tissue

For both leaves and flowers, three replicates were pooled to make one representative sample. Leaves and flower tissues were subjected to aqueous methanol metabolite extraction. Metabolic profiles were obtained using reverse phase liquid chromatography combined with a quadrupole time of flight mass spectrometer (LC-QTOF-MS) (De Vos et al. 2007). Metabolite profiles obtained were processed using MetAlign software (Lommen 2009). MSClust software (Tikunov et al. 2012) was used for clustering masses that originate from the same parent ion. Qualitative variation between the parents and the epiRILs was assessed using the selected-ion monitoring chromatogram. Quantitative variation was analyzed using the total ion count (see Supplemental Material for details on extraction methods and run parameters).

### KO analysis by UPLC-Orbitrap-FTMS

The mutant samples were analyzed on a Waters UPLC-PDA connected to an LTQ Orbitrap-FTMS hybrid system (Van Duynhoven et al. 2014). Five times three pooled samples of flower heads were analysed per line. Aqueous-methanol extracts from KO and WT control plants were analyzed using the same chromatographic conditions as in the LC-QTOF MS analyses described above. Metabolite profiles obtained were processed using the same MetAlign-MSClust-based workflow as described above. Identification of metabolites was based on matching the retention time and accurate masses of parent ions and their (in-source) fragments with an in-house experiment-based database of previously reported *Arabidopsis* metabolites, detected under the same chromatographic conditions (Van Der Hooft et al. 2012). Compounds not present in this in-house database were matched with molecular ion masses of compounds present in other open databases such as the Dictionary of Natural Products (http://dnp.chemnetbase.com), HMDB (http://www.hmdb.ca), and KNApSAcK (http://kanaya.naist.jp/knapsack_jsp/top.html). Compounds were given a metabolite identification level according to the Metabolomics Society Initiative (MSI) (Summer et al. 2007). Before statistical analysis, metabolite intensities were log_10_-transformed (see Supplemental Material for details on extraction methods and run parameters).

### Epigenetic QTL mapping with R/QTL

Quantitative variation in metabolite accumulation was assessed using the total ion count, and mass clusters were batch-corrected by dividing the metabolite sample intensity by the metabolite intensity batch average. The batch-corrected values for the epiRILs were used for QTL^epi^ mapping. To control for the effect of flowering time on the metabolic trait values, a parallel analysis was run where the metabolic trait values were divided by flowering time for each specific epiRIL. Epigenetic QTL mapping was performed with multiple QTL mapping (MQM) implemented in the R/QTL software (Arends et al. 2010; Joosen et al. 2012). Cofactors were assigned to 42 of the 126 markers based on their physical cM position and preliminary composite interval mapping (CIM) on the data. Backward elimination was used to remove cofactors that did not contribute to the fit of the model. MQM mapping was performed on each trait and each treatment separately, and the results were compared to standard interval mapping, using Haley Knott regression (Haley and Knott 1992). One thousand random permutations were generated for each phenotype to determine the LOD significance threshold with α = 0.05 as the genome-wide type I error level (see Supplemental Material for software settings).

### Calculation of methylation scores

Probe-level methylation data were obtained for 89 epiRILs of this study from the MeDIP tiling arrays as in Cortijo et al. (2014). The methylation calls were previously determined for each probe on these arrays using a Hidden Markov Model (Colome-Tatche et al. 2012). Based on these results, posterior probability for probe *i* to be unmethylated or methylated was calculated by *post*(*P*_*i*_ = *U*) and *post*(*P*_*i*_ = *M*), respectively. Using this, the methylation level of probe I was defined as *ML* –*post*(*P*_*i*_ = *U*)*(−1) + *post*(*P*_*i*_ = *M*)*1 (for further details, see Cortijo et al. 2014). Scores between −1 and −0.3 were counted as hypomethylated; scores between −0.3 and 1 were counted as methylated.

### Small RNA target gene selection

To account for the possibility of RdDM activity, a set of methylomes captured with MeDIP-ChIP technology for a population of 123 epiRILs and their parental lines (Cortijo et al. 2014) were utilized in a search for genomic loci containing probes that meet the following criteria: (1) They fall inside a gene promoter; (2) the variance for the methylation calls across the population of epiRILs is >0; (3) parental lines are polymorphic in terms of DNA methylation; (4) they have at least two consecutive probes that correlate with a metabolite-associated QTL^epi^ peak marker; and (5) the genes are located outside the QTL interval with which significant correlation was determined. The promoters of such genes were further subjected to a search for segments sharing perfect similarity with DNA regions inside the related QTL^epi^. These regions were then decomposed, in silico, into sets of artificial sRNAs, which were then submitted to the SAILS framework to predict the loading to AGO4/6/9 proteins. Finally, the artsRNAs were matched to true sRNAs from wild-type (WT) and *ddm1* sRNA libraries (Slotkin et al. 2009; see Supplemental Material for details on selection criteria and the SAILs framework).

### Quantitative real-time PCR

RNA was extracted for 93 epiRILs using the Direct-zol RNA MiniPrep Kit from Zymo Research. Remaining DNA was removed using RQ1 RNase-free DNase (Promega). cDNA synthesis was performed using the iScript cDNA Synthesis Kit (Bio-Rad). The RT-PCR was performed on the CFX96 (Bio-Rad). The primers used are listed in Supplemental Table S10. Genes were normalized against the reference genes *SAND* and *TIP41* (see Supplemental Material for details on PCR settings).

### Whole-genome bisulphite sequencing (WGBS)

WGBS data for four epiRILs (epiRIL92, epiRIL150, epiRIL193, and epiRIL232) were obtained from Lauss et al. (2018) and reanalyzed for the earlier reported DMR markers (Cortijo et al. 2014) to confirm the stability of the epiRILs. In brief, aerial rosette tissue at 21/22 DAS (days after sowing) was harvested and snap-frozen immediately in liquid nitrogen. Material was stored at −80°C until processing. Genomic DNA from two biological replicates (2 × 6 rosettes) was extracted using a standard CTAB-based extraction protocol followed by an RNase digest. Five micrograms of DNA per sample was submitted to BGI for bisulphite treatment, library construction (insert size of 200 bp), and sequencing. Sequencing (whole-genome bisulphite sequencing) was performed on an Illumina HiSeq 4000 instrument, generating 150-bp paired-end reads.

## Data access

Mass spectrometry data of epiRILs and knockout mutants are available from the DRYAD Digital Repository (https://doi.org/10.5061/dryad.ph37b2q). Bisulphite sequencing data of epiRILs from this study have been submitted to the Gene Expression Omnibus (GEO; https://www.ncbi.nlm.nih.gov/geo/) under accession number GSE122398.

## Supplementary Material

Supplemental Material
