# Supplementary material for: Epigenetic mapping of the Arabidopsis metabolome reveals mediators of the epigenotype-phenotype map
Source: Genome Res. 2019 Jan;29(1):96–106. doi: 10.1101/gr.232371.117 (PMC6314165; doi:10.1101/gr.232371.117)
Supplement: Supplemental Material [file supp_29_1_96__index.html]

Epigenetic mapping of the Arabidopsis metabolome reveals mediators of the epigenotype-phenotype map — Epigenetic mapping of the Arabidopsis metabolome reveals mediators of the epigenotype-phenotype map — Supplemental Material 

# Epigenetic mapping of the *Arabidopsis* metabolome reveals mediators of the epigenotype-phenotype map

## Supplemental Material

- Supplemental\_Material.docx
- Supplemental\_Tables.xlsx
