# Supplementary material for: Epigenetic mapping of the Arabidopsis metabolome reveals mediators of the epigenotype-phenotype map
Source: Genome Res. 2019 Jan;29(1):96–106. doi: 10.1101/gr.232371.117 (PMC6314165; doi:10.1101/gr.232371.117)
Supplement: Supplemental Material [file supp_gr.232371.117_Supplemental_Material.docx]

**Supplemental Material**

**Supplemental Methods**

**Plant growth conditions**

Seeds from 96 epiRILs and their parents, Col-0 and *ddm1-2,* all in the *Arabidopsis thaliana* Col-0 background were obtained from the INRA Arabidopsis Resource Center in Versailles (<http://dbsgap.versailles.inra.fr/portail/>). Seeds of the epiRILs were derived from the bulk propagation of one of the three epiRIL BC 1-S6 [F_8_] sublines described in (Johannes et al, 2009 PLoS Genet 5: e1000530). Seeds of Col wild type and the *ddm1-2* mutant were of the 5^th^ generation. All seeds were sown on filter paper with demi water and stratified at 4˚C in darkness for 5d. Subsequently, seeds were transferred to a culture room (16h light, 24˚C) to induce seed germination for 42h. Seventeen replicates per epiRIL and parent were completely randomly transplanted to wet Rockwool blocks of 4 x 4 cm in a climate chamber (16h light, 125 µmolm^-2^s^-1^, 70% RH, 20/18˚C day/night cycle). All plants were watered every morning for 5 min at 9am with 1/1000 Hyponex solution (Hyponex, Osaka, Japan). At 21 days after germination (DAG), six randomly selected replicates were harvested for leaf tissue. At the time of flowering, the flower head was harvested for six other randomly selected replicates.

**KO analysis**

Homozygous mutants (Med-8 (M1; AT2G03070), SALK_202443C (S1; AT1G50740), SALK_032288C (S2; AT3G24360)) were ordered via the Nottingham Arabidopsis Stock Centre (NASC) and checked for homozygosity in a first experiment. After confirmation, wild-type and mutant seeds were harvested. Twenty-seven replicates per line were grown in a completely randomized design in exactly the same conditions as listed above (Plant growth conditions). For all replicates, flowering time was noted at the opening of the first flower in the morning. For 15 replicates, flower heads were harvested in the late afternoon when the first flower had opened in the morning. Plants were photographed from above for the entire growth period (until leaves started to overlap). For the twelve remaining replicates, main stem branching (MSB), rosette branching, plant height at 1^st^ silique (PH1S), total plant height (TPH) and average internode length (AIL) were measured two weeks after flowering. Gene expression of KO lines was analyzed using qPCR. Gene expression was completely aborted in S1 and M1, while it was reduced by a factor of five in S2 (Supplemental figure 2).

**LC-QTOF-MS analysis of leaf and flower tissue**

For both leaves and flowers, three replicates were pooled to make one representative sample. The other eleven replicates were analyzed for morphological differences, described elsewhere (Kooke et al., 2015). For the leaves, 0.05 g tissue was grinded and extracted using 200 μL aqueous methanol (methanol (94%), formic acid (0.125%) and demi-water) in 1.5 ml Eppendorf tubes. For the flower tissue, between 0.015 and 0.06 g tissue, depending on the sample, was grinded and extracted using tissue/methanol (methanol (94%), formic acid (0.125%) and demi-water) 0.01 g/100 μL, proportionally in 1.5 ml Eppendorf tubes. After addition of methanol to the frozen plant powder, the Eppendorf tubes were immediately vortexed. Subsequently, all samples were sonicated for 15 min and then centrifuged for 10 min. Supernatant was vacuum filtrated using 96-well protein filtration plates (Captiva 0.45mm, Ansys Technologies) and collected in 700 µl glass cuvettes in 96-well autosampler plates (Waters) using a Genesis work system (Tecan Systems).

Metabolic profiles of the prepared methanol extracts were obtained using reverse phase liquid chromatography combined with a quadrupole time of flight high-resolution mass spectrometer (LC-QTOF-MS) (De Vos et al., 2007). In short, 5µl of extract was injected in an Alliance 2796 HPLC system equipped with a Luna C18 (150 x 2.0 mm, 3 µm) column (Phenomenex). Separation was performed using a linear gradient of 5% acetonitrile in ultrapure water (both acidified with 0.1% formic acid) to 35% acetonitrile in 45 minutes at a flow rate of 0.19 ml min^-1^. After detection of the compounds eluting from the column with a photodiode array detector (PDA; 200-700 nm), negative electrospray ionization was applied and masses in the range of m/z 80 to 1500 were detected in a QTOF Ultima MS (Waters) at a mass resolution of 10,000.

Metabolite profiles obtained were processed using MetAlign software (Lommen, 2009) for baseline correction, noise estimation and ion wise mass spectral alignment. This resulted in 8955 mass signals for leaf samples and 6738 mass signals for flower samples. All masses with amplitudes above 100 in at least 10 samples were kept, resulting in 2334 masses for leaf samples and 1818 masses for flower samples. MSClust software (Tikunov et al., 2012) was used for clustering masses that originate from the same parent ion based on their corresponding retention times and intensity patterns over samples. In the end, 216 mass clusters representing reconstructed metabolites were obtained for leaf samples and 179 for flower samples. Qualitative variation between the parents and the epiRILs was assessed using the selected-ion monitoring chromatogram. Quantitative variation was analyzed using the total ion count.

**KO analysis by UPLC-Orbitrap-FTMS**

The mutant samples were analyzed on a Waters UPLC-PDA connected to an LTQ Orbitrap-FTMS hybrid system. At the time of flowering, flower heads were harvested and three replicates were pooled to make one representative replicate sample (five replicates per WT/ KO). Aqueous-methanol extracts were prepared from 30 to 50 mg frozen ground material to which 120 to 200 µl (depending on the fresh weight of sample) of 80% MeOH containing 0.1% formic acid was added (De Vos et al., 2007). Samples were immediately vortexed, sonicated for 15 min and then centrifuged for 15 min.

The crude extracts from KO and WT control plants were analyzed using an UHPLC (Waters Aquity) coupled to a high-resolution LTQ Orbitrap FTMS (Thermo) using the same chromatographic conditions as in the LC-QTOF MS analyses described above and negative ionization mode; details of Orbitrap MS setting are described elsewhere (van Duynhoven et al., 2014). Metabolite mases were detected in negative electrospray ionization mode at a mass resolution of 60,000 .

Metabolite profiles obtained were processed in an untargeted manner using the same Metalign-MSClust based workflow as described above. In the end, 249 mass clusters representing putative metabolites were obtained.

Identification of metabolites was based on matching the retention time and accurate masses of parent ions and their (in-source) fragments with an in-house experiment-based database of previously reported Arabidopsis metabolites detected under the same chromatographic conditions (van der Hooft et al., 2012) (MSI: Supplemental table 10). Compounds not present in this in-house database were matched with molecular ion masses of compounds present in other open databases such as the Dictionary of Natural Products (<http://dnp.chemnetbase.com>), HMDB (<http://www.hmdb.ca>) and KNApSAcK (<http://kanaya.naist.jp/knapsack_jsp/top.html>). Compounds were given a metabolite identification level according to Metabolomics Society Initiative (MSI; Summer et al., 2007) Supplemental table 10). Before statistical analysis, metabolite intensities were log_10_-transformed.

**Epigenetic QTL mapping with R/QTL**

Quantitative variation in metabolite accumulation was assessed using the total ion count and mass clusters were batch corrected by dividing the metabolite sample intensity by the metabolite intensity batch average. The batch corrected values for the epiRILs were used for QTL^epi^ mapping. To control for the effect of flowering time on the metabolic trait values, a parallel analysis was run where the metabolic trait values were divided by flowering time for each specific epiRIL. Epigenetic QTL mapping was performed with multiple QTL mapping (MQM) implemented in the R/QTL software (Joosen et al., 2012, Arends et al., 2010). Co-factors were assigned to 42 out of the 126 markers based on their physical cM position and preliminary composite interval mapping (CIM) on the data. Backward elimination was used to remove cofactors that did not contribute to the fit of the model. MQM mapping was performed on each trait and each treatment separately and the results were compared to standard interval mapping, using Haley Knott regression (Haley and Knott, 1992). Thousand random permutations were generated for each phenotype to determine the LOD significance threshold with 0.05 as the genome-wide type I error level.

**Calculation of methylation scores**

Probe-level methylation data was obtained for 89 epiRILs of this study from the MeDIP tiling arrays as in (Cortijo et al., 2014). The methylation calls were previously determined for each probe on these arrays using a Hidden Markov Model (Colome-Tatche et al., 2012). Based on these results, posterior probability for probe *i* to be un-methylated or methylated was calculated by *post*(*P_i_ = U*) and *post*(*P_i_ = M*), respectively. Using this, the methylation level of probe I was defined as *ML –post*(*P_i_ = U*)*(-1) + *post*(*P_i_ = M*)*1 (For further details, see (Cortijo et al., 2014)). Scores between -1 and -0.3 were counted as hypo-methylated, scores between -0.3 and 1 were counted as methylated.

**Small RNA target gene selection**

To account for the possibility of RdDM activity, a set of methylomes captured with MeDIP-ChiP technology for a population of 123 epiRILs and their parental lines (Cortijo et al., 2014) were utilized in a search for genomic loci containing probes that verify the following conditions: (i) fall inside a gene promoter (For all analysis in this manuscript 1500 bp upstream of the transcriptional start site is considered the promotor region); (ii) the variance for the methylation calls across the population of epiRILs is > 0; (iii) parental lines are polymorphic in terms of DNA methylation (i.e. WT is methylated and *ddm1* is unmethylated); (iv) have at least 2 consecutive probes (one "mismatch" allowed) that correlate with a metabolite-associated QTL^epi^ peak marker (Spearman’s correlation, FDR<0.1) in terms of methylation scores across the epiRIL population; (v) the genes are located outside the QTL interval with which significant correlation was determined.

By applying these filters, a set of 324 candidate target genes were identified. The promoters of such genes were further subjected to a search for segments sharing perfect similarity with DNA regions inside the related QTL^epi^. These regions were then decomposed, in silico, into sets of artificial sRNAs (artsRNAs) with a length in the range 21-24nt to simulate candidate sRNA sequences that can map to the gene and the QTL^epi^ interval. The artificial sRNAs (artsRNAs) found where then submitted to the SAILS framework to predict the possibility of being loaded to AGO4/6/9 proteins and therefore assume a transcriptional silencing role in the plants. Those genes that did not contain artsRNA showing the sequence properties necessary for loading into the proper AGOs were discarded.

Finally, the remaining artsRNAs were matched to true sRNAs from wild-type (WT) and *ddm1* sRNA libraries (Slotkin et al., 2009) to obtain further evidence to support these segments as real sRNAs.

**SAILS framework**
SAILS is a computational tool for biologically functional sRNA detection and categorization based on AGO-sRNA affinity. During a development phase, a machine learning approach based on Support Vector Machines was employed to identify sRNA sequence patterns from deeply sequenced sRNAs after immunoprecipitation of AGO proteins from *Arabidopsis thaliana*. Optimized feature sets were then used to build an inference system composed of 3 layers intended to classify sRNA sequences according to their AGO affinity: the 1st layer includes a binary SVM model that filters out sequences that do not show strong evidence to gather the properties necessary to binding to AGO proteins and that therefore are expected to be inactive; the 2nd layer is composed by an ensemble of binary one-vs-one classifiers, each trained to explore the dissimilarities in AGO-bound sRNA sequences in a pairwise fashion; and finally, the 3rd layer comprises a voting system that assigns scores to each AGO-sRNA possibility using the decision values produced in the previous layer.
 
A data set covering several genome-wide deep-sequenced wild-type (WT) plants and AGO-IP sRNA libraries was gathered for training, testing and validation purposes. The sets used for the development of the inference system were acquired from the same ecotype plants and comparable tissue composition, and included a WT library and 8 AGO-IP libraries (AGOs: 1, 2, 4, 5, 6, 7, 9 and 10). More than 40 other sRNA libraries were recycled in a validation phase, comprising AGO-IP data sets from different tissues, as well samples collected from pathogen infected plants and lists of siRNA from public databases experimentally validated, namely microRNAs and trans-acting sRNAs.
The classifiers demonstrated high accuracy in distinguishing AGO-bound sRNA against other sequences, reaching an accuracy of nearly 80% in a balanced test set. For individual AGO-specific models, accuracy was on average 60%, providing clade predictions with an accuracy of 85% and assigning correctly functional groups (transcriptional or post-transcriptional activity) with 96% accuracy.

**Quantitative Real-Time PCR**

RNA was extracted for 93 epiRILs using the Direct-zol RNA miniprep kit from Zymo research. Remaining DNA was removed using RQ1 RNase-free DNase (Promega). cDNA synthesis was performed using the iScript cDNA synthesis Kit (Bio-Rad). For each qPCR, 3 µl of sample, 5 µl of iQ SYBR Green Supermix (Bio-Rad) and 1 µl of each primer (10 mM) were mixed. The RT-PCR was performed on the CFX96 (Bio-Rad). The program was started with a cycle of 95°C for 3 min and then 35 cycles of 10 s at 95°C, 30 s at 53°C and 30 s at 72°C followed by a melt curve cycle starting from 65°C to 95°C, raising the temperature by 0.5°C every 5 s. The primers used are listed in Supplemental table 9. Genes were normalized against the reference genes SAND and TIP41. Although both reference genes gave comparable results, the results presented use TIP41 as reference gene.

**Whole genome bisulphite sequencing (WGBS)**

WGBS data for four epiRILs (epiRIL92, epiRIL150, epiRIL193 and epiRIL232) were obtained from Lauss et al. (2018) and re-analyzed for the earlier reported DMR markers (Cortijo et al., (2014)) to confirm the stability of the epiRILs. In brief, aerial rosette tissue at 21/22 DAS (days after sowing) was harvested and snap-frozen immediately in liquid nitrogen. Material was stored at –80°C until processing. Genomic DNA from two biological replicates (2 x 6 rosettes) was extracted using a standard CTAB-based extraction protocol followed by an RNase digest. 5μg DNA per sample was submitted to BGI for Bisulphite treatment, library construction (insert size of 200 bp) and sequencing. Sequencing (whole-genome bisulphite sequencing; WGBS-seq) was performed on an Illumina HiSeq 4000 instrument, generating 150 bp paired-end reads.

**Supplemental figures**


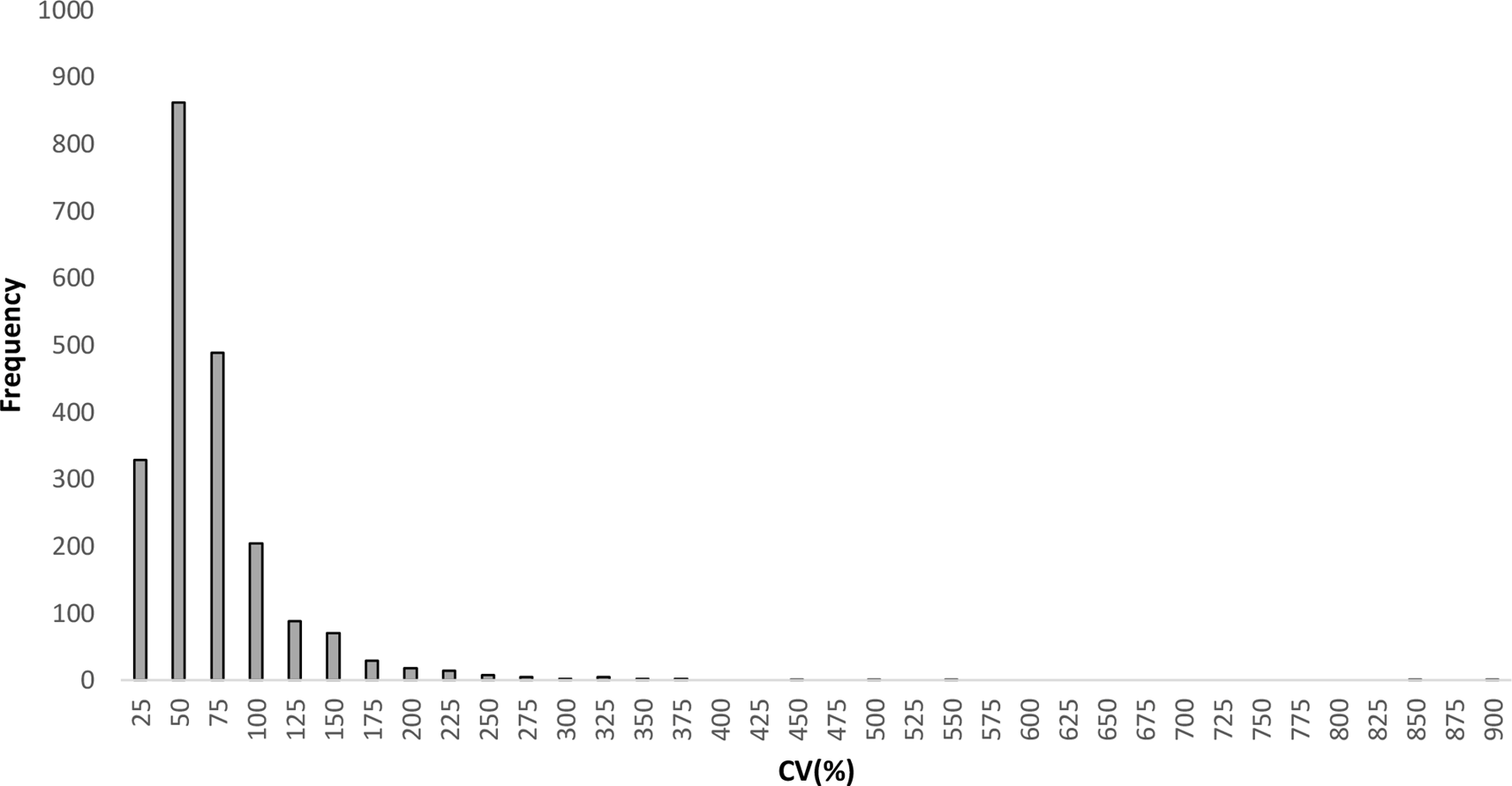


**Supplemental figure 1.** Frequency distribution of coefficient of variation (%) of mass peaks of secondary metabolites detected in the Cvi x Ler RIL population using untargeted LC-QTOF-MS-based metabolomics (Keurentjes et al., 2006).


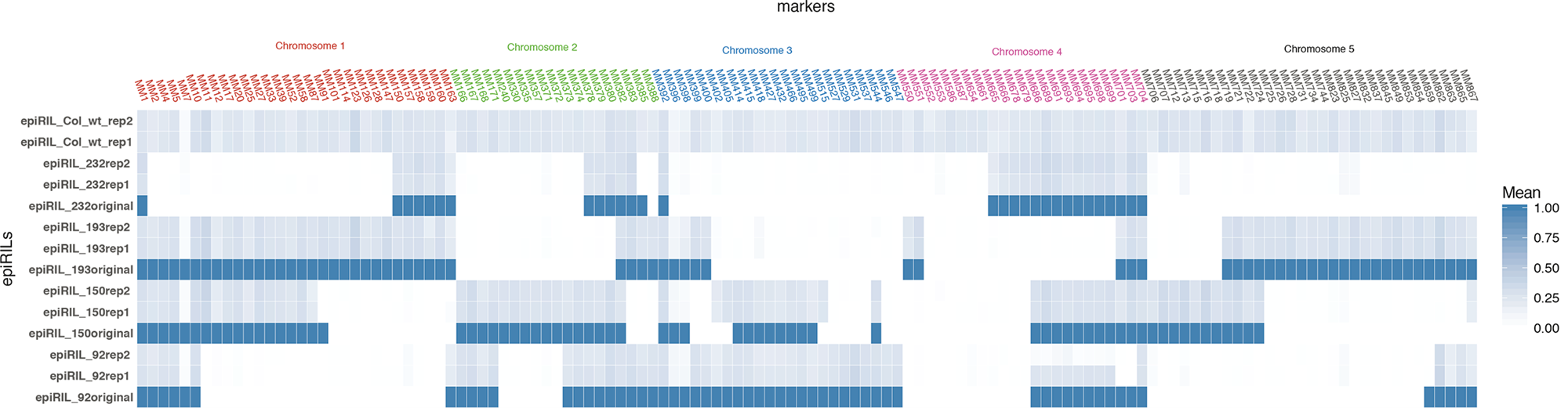


**Supplemental figure 2.** Methylation level of DMRs in Col-0 WT, epiRIL92, epiRIL150, epiRIL193 and epiRIL232 measured with whole genome bisulphite sequencing (Lauss et al., 2018). Two replicates were used for each epiRIL and compared to the original DMRs as reported in Cortijo et al. (2014).


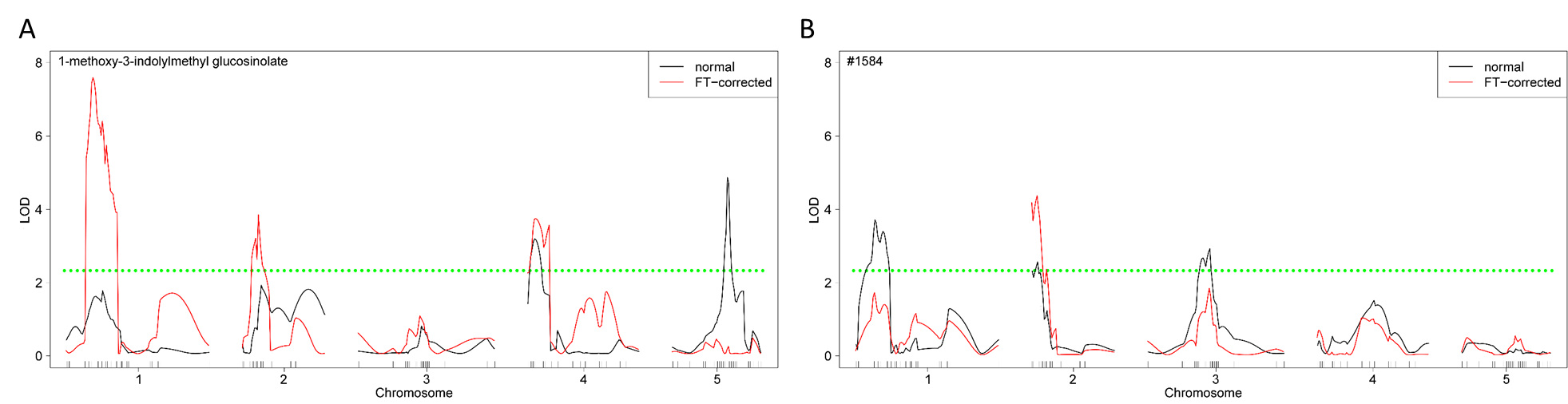


**Supplemental figure 3.** QTL^epi^ plots for (A) flower metabolite 1 methoxy-3-indolylmethyl glucosinolate and (B) leaf metabolite #1584. The black line depicts the QTL^epi^ plot for uncorrected metabolite values, while the red line depicts the QTL^epi^ plot for flowering time-corrected metabolite values. The green dashed line is the QTL^epi^ – threshold.


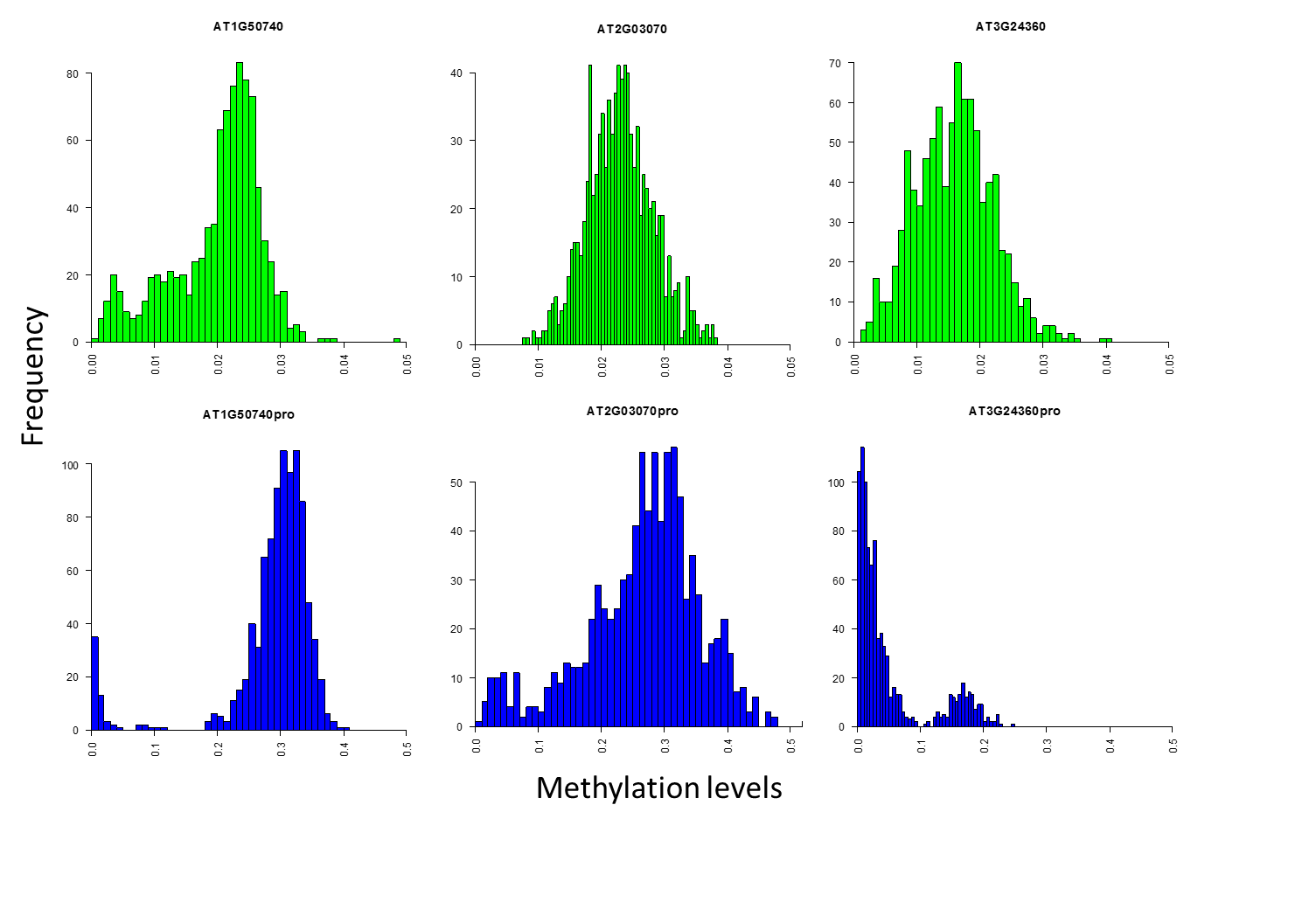


**Supplemental figure 4.** Methylation levels of the three candidate genes AT1G50740, AT2G03070 and AT3G24360 in natural accessions of Arabidopsis thaliana. Methylation levels in the gene body are displayed in green, methylation levels in the promoter region are displayed in blue. Data are obtained from the Arabidopsis 1001 genome project (http://neomorph.salk.edu/1001.aj.php).


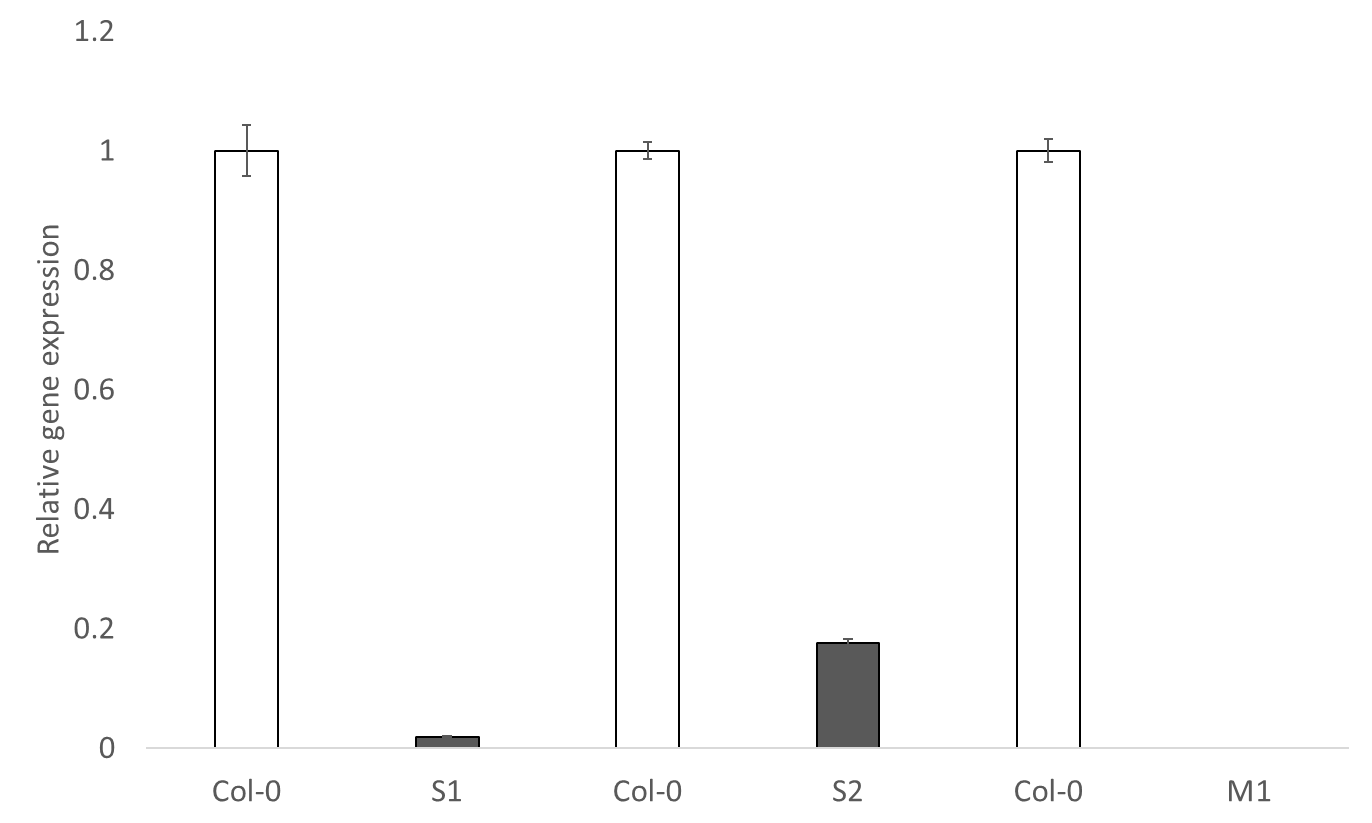


**Supplemental figure 5.** Relative gene expression of AT1G50740 in Col-0 and SALK1 (S1), AT3G24360 in Col-0 and SALK2 (S2), and AT2G03070 in Col-0 and med-8 (M1) measured with qPCR.


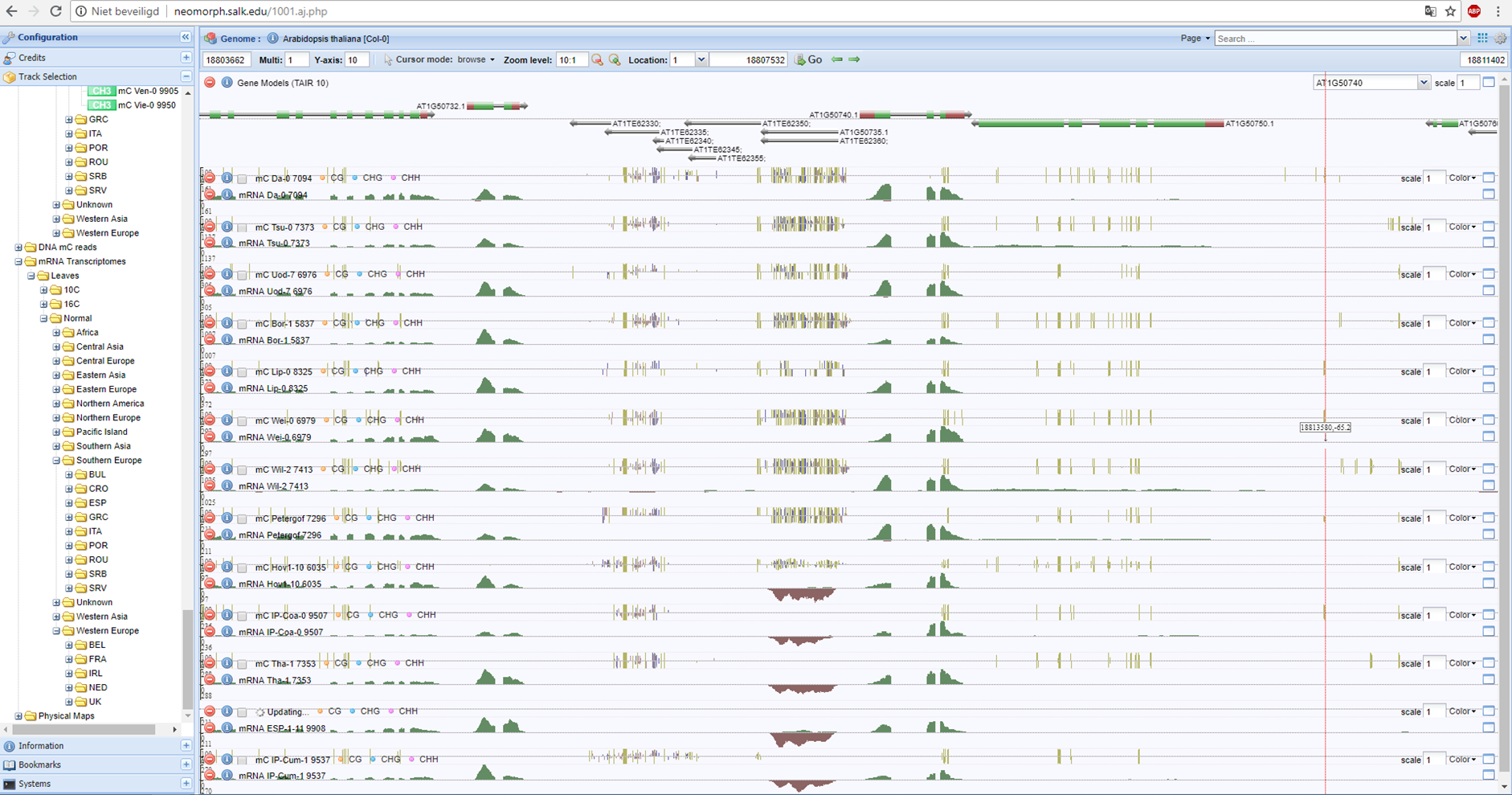


**Supplemental figure 6A.** Screenshot of the methylation level and gene expression of AT1G50740 in natural accessions of Arabidopsis thaliana (neomorph.salk.edu/1001.aj.php). 13 Accessions are displayed as a representative example. For each accession methylation is displayed with yellow (CG), blue (CHG) and purple (CHH) vertical lines, under which gene expression is given in red (-) and green (+).


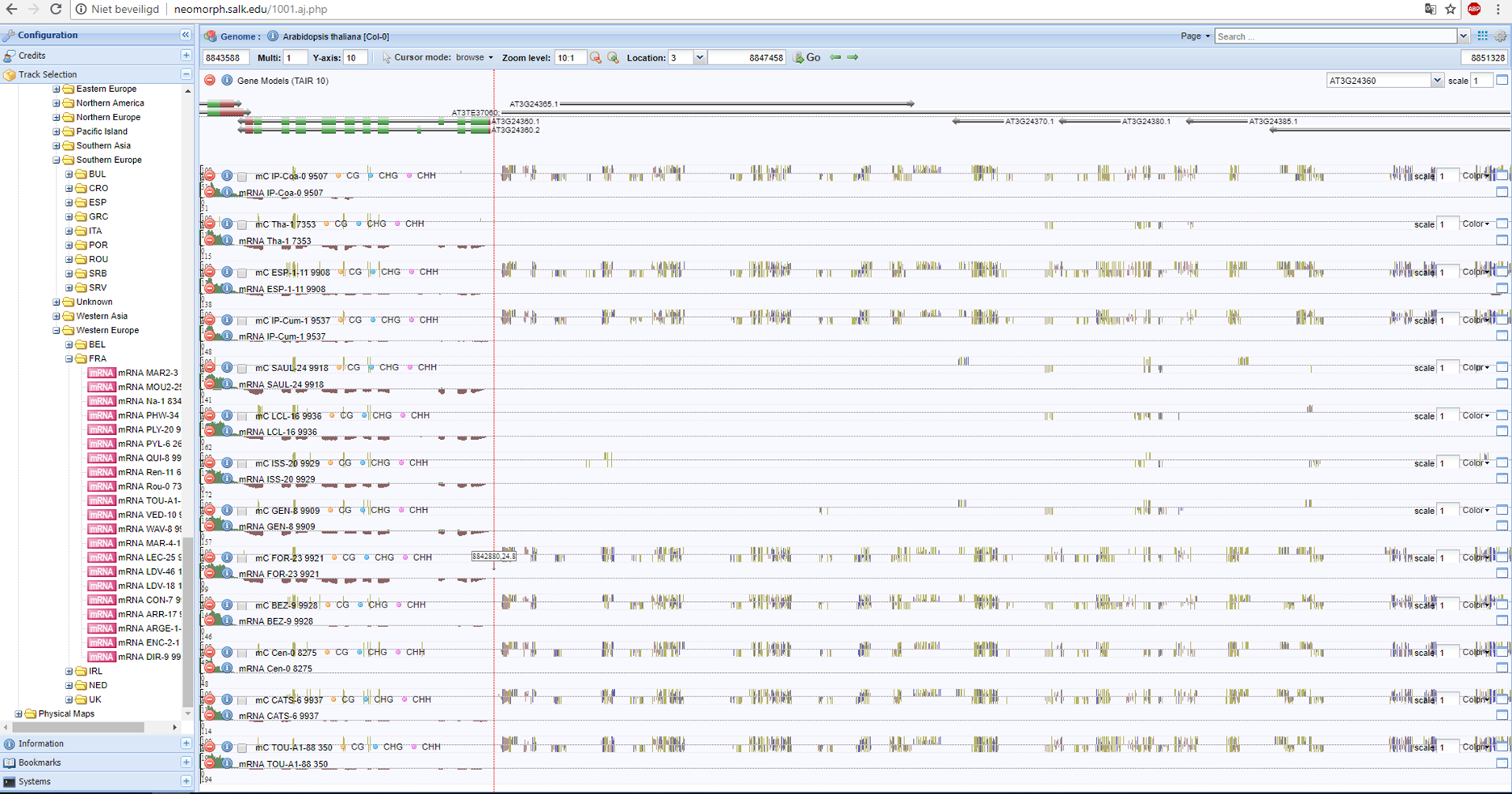


**Supplemental figure 6B.** Screenshot of the methylation level and gene expression of AT3G24360 in natural accessions of Arabidopsis thaliana (neomorph.salk.edu/1001.aj.php). 13 Accessions are displayed as a representative example. For each accession methylation is displayed with yellow (CG), blue (CHG) and purple (CHH) vertical lines, under which gene expression is given in red (-) and green (+).


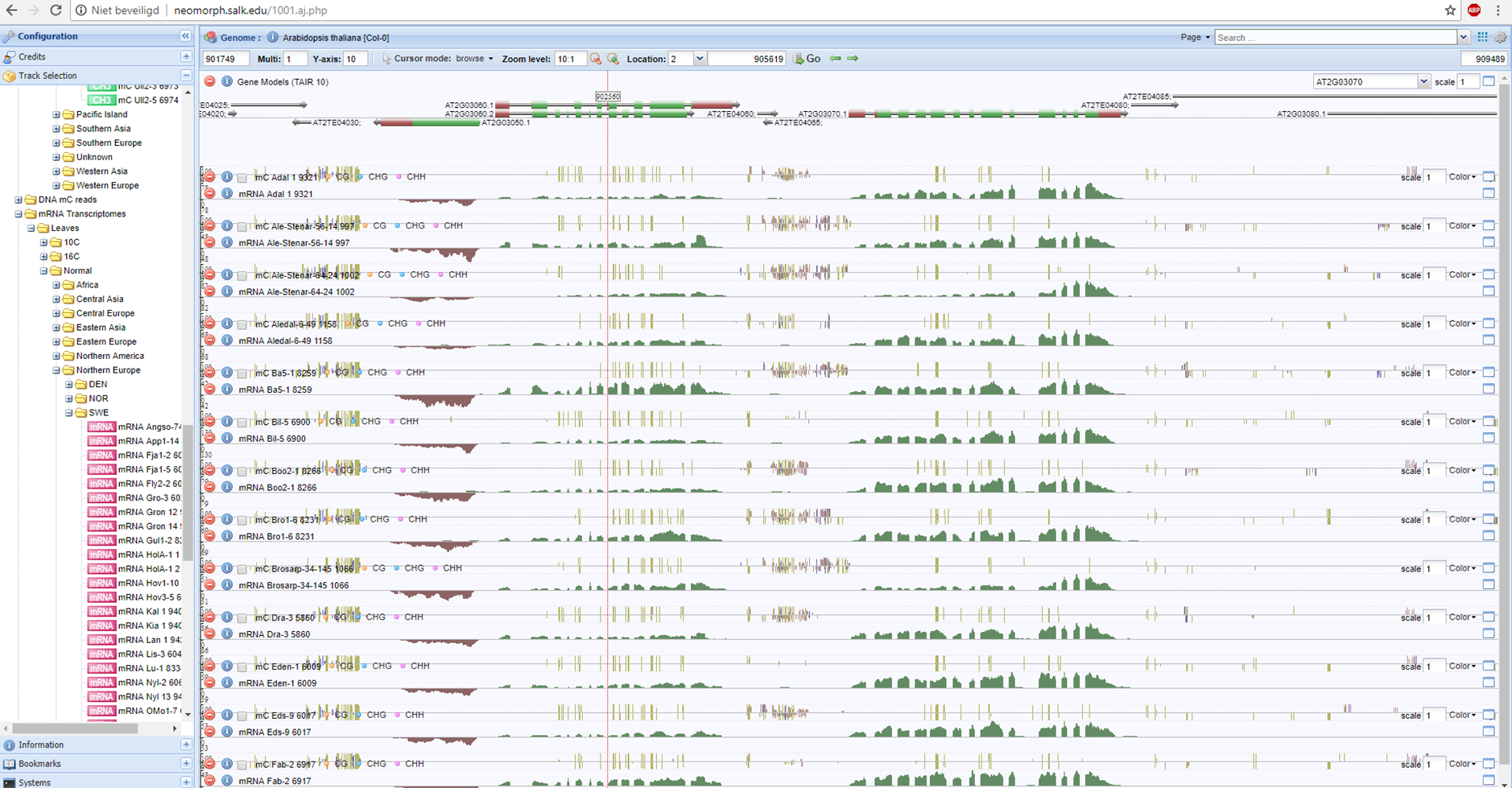


**Supplemental figure 6C.** Screenshot of the methylation level and gene expression of AT2G03070 in natural accessions of Arabidopsis thaliana (neomorph.salk.edu/1001.aj.php). 13 Accessions are displayed as a representative example. For each accession methylation is displayed with yellow (CG), blue (CHG) and purple (CHH) vertical lines, under which gene expression is given in red (-) and green (+).
